# Pyrotinib-Containing Neoadjuvant Therapy in Patients With HER2-Positive Breast Cancer: A Multicenter Retrospective Analysis

**DOI:** 10.3389/fonc.2022.855512

**Published:** 2022-04-07

**Authors:** Xiaoyun Mao, Pengwei Lv, Yiping Gong, Xiujuan Wu, Peng Tang, Shushu Wang, Dianlong Zhang, Wei You, Ouchen Wang, Jun Zhou, Jingruo Li, Feng Jin

**Affiliations:** ^1^ Department of Breast Surgery, The First Affiliated Hospital of China Medical University, Shenyang, China; ^2^ Department of Breast Surgery, The First Affiliated Hospital of Zhengzhou University, Zhengzhou, China; ^3^ Department of Breast, Renmin Hospital of Wuhan University, Hubei General Hospital, Wuhan, China; ^4^ Department of Breast and Thyroid Surgery, The Southwest Hospital of Army Medical University, Chongqing, China; ^5^ Department of Breast Surgery, Dalian Municipal Central Hospital, Dalian, China; ^6^ First Department of Breast Surgery, Henan Provincial People’s Hospital, Zhengzhou, China; ^7^ Department of Breast Surgery, The First Affiliated Hospital of Wenzhou Medical University, Wenzhou, China; ^8^ Department of Thyroid and Breast Surgery, The First People’s Hospital of Lianyungang, Lianyungang, China; ^9^ Second Department of Breast Surgery, The First Affiliated Hospital of Zhengzhou University, Zhengzhou, China

**Keywords:** human epidermal growth factor receptor 2, breast cancer, pyrotinib, neoadjuvant, real-world

## Abstract

**Background:**

Pyrotinib, a small-molecule tyrosine kinase inhibitor, has been investigated as a component of neoadjuvant therapy in phase 2 trials of human epidermal growth factor receptor 2 (HER2)-positive breast cancer. This study aimed to evaluate the effectiveness and safety of pyrotinib-containing neoadjuvant therapy for patients with HER2-positive early or locally advanced breast cancer in the real-world setting.

**Methods:**

Data of 97 patients with HER2-positive breast cancer from 21 centers across China treated with pyrotinib-containing neoadjuvant therapy were reviewed. Neoadjuvant therapy consisted of taxane/carboplatin/trastuzumab plus pyrotinib (TCbH+Py, 30 [30.9%]), anthracycline/cyclophosphamide followed by taxane/trastuzumab plus pyrotinib (AC-TH+Py) or taxane followed by anthracycline/cyclophosphamide/trastuzumab plus pyrotinib (T-ACH+Py, 29 [29.9%]), taxane/trastuzumab plus pyrotinib (TH+Py, 23 [23.7%]), and other pyrotinib-containing neoadjuvant treatment (15 [15.5%]). The primary outcome was breast pathological complete response (bpCR, ypT0/is) rate. Secondary outcomes included total pathological complete response (tpCR, ypT0/is ypN0) rate, objective response rate (ORR), and the incidence of preoperative adverse events.

**Results:**

The ORR of pyrotinib-containing neoadjuvant therapy was 87.6% (85/97). The bpCR and tpCR rates were 54.6% (95% confidence interval [CI], 44.2%-64.7%) and 48.5% [95% CI, 38.2%-58.8%], respectively. The most common grade 3 or 4 treatment-related adverse events included diarrhea (15 [15.5%]), decreased hemoglobin (nine [9.3%]), and decreased neutrophil count (eight [8.2%]). No treatment-related deaths occurred.

**Conclusion:**

Pyrotinib-containing neoadjuvant therapy for patients with HER2-positive early or locally advanced breast cancer shows favorable effectiveness with manageable toxicity in the real-world setting. Trastuzumab plus pyrotinib may be a novel option of dual HER2-targeted blockade.

## Introduction

Breast cancer is the most common malignant tumor in women. Approximately 10%-13% of women will develop breast cancer during their lifetime (1). Human epidermal growth factor receptor 2 (HER2)-positive breast cancer accounts for 15%-20% of all breast cancers (2). HER2-targeted therapy dramatically improved outcomes in HER2-positive breast cancer. Chemotherapy combined with dual HER2-targeted blockade has become a standard neoadjuvant regimen (3–8). Actually, more than half of patients can achieve pathological complete response (pCR) with neoadjuvant chemotherapy plus dual HER2-targeted monoclonal antibodies (trastuzumab and pertuzumab) (9–11). A randomized, open-label phase 3 KRISTINE trial showed a pCR rate of 44.4% (99 of 223) with trastuzumab emtansine plus pertuzumab and 55.7% (123 of 221) with docetaxel, carboplatin, and trastuzumab plus pertuzumab (absolute difference, -11.3%; 95% confidence interval [CI], -20.5% to -2.0%; P=0.016) (9). Chemotherapy combined with trastuzumab and a small-molecule tyrosine kinase inhibitor (TKI, such as lapatinib and neratinib) could also result in favorable breast pCR (bpCR) rate (51.3%-62.0%) (12–16) with acceptable safety profile. A meta-analysis demonstrated that pCR was associated with substantially longer event-free survival and overall survival in HER2-positive breast cancer (17). Thus, the dual HER2-targeted blockade with a macromolecular monoclonal antibody and a small-molecule TKI as components of neoadjuvant therapy deserves further investigation.

Pyrotinib is a small-molecule, irreversible TKI, targeting HER1, HER2, and HER4 (18). The phase 3 PHOEBE study confirmed the superiority of pyrotinib over lapatinib when combined with capecitabine in the treatment of HER2-positive relapsed or metastatic breast cancer, with significantly better objective response rate (ORR, 67.2% vs. 51.5%) and progression-free survival (12.5 months vs. 6.8 months) (19). Pyrotinib as a component of HER2-targeted neoadjuvant therapy has been investigated in small phase 2 clinical trials (20–22). We did a multicenter retrospective analysis to assess effectiveness and safety of pyrotinib-containing regimens as neoadjuvant therapy for patients with HER2-positive breast cancer in the real-world setting.

## Methods

### Study Design and Participants

This was a retrospective, observational real-world study of female adult patients with HER2-positive breast cancer who received pyrotinib-containing neoadjuvant therapy and surgery at 21 centers (**Supplementary Table S1**) across China between November 2018 and January 2021. All eligible patients should have histopathologically confirmed stage II-III HER2-positive (immunohistochemistry score of 3+, or 2+ with gene amplification by fluorescence *in-situ* hybridization) invasive breast cancer, with at least one measurable lesion according to the Response Evaluation Criteria In Solid Tumors (RECIST) version 1.1. Pyrotinib must be used for at least one neoadjuvant cycle. Patients who received other neoadjuvant HER2-TKIs were excluded. This study was approved by the ethics committee of the First Hospital of China Medical University (No. [2021]308). Informed consent was waived due to the retrospective nature of the study.

### Treatment

The standard dose of pyrotinib was 400 mg once a day. The combination regimen of neoadjuvant therapy and the initial dose of pyrotinib were at the discretion of local investigator. Dose reduction, interruption, and discontinuation of pyrotinib were allowed according to the adverse events (AEs).

### Data Collection

Demography, baseline disease characteristics, information of neoadjuvant therapy and surgery, imaging and pathological results, and safety data were all extracted from the medical records. Radiographic images were assessed by the local investigator according to RECIST 1.1. Pathological reports were completed by the local pathologist.

### Outcomes

The primary outcome was bpCR rate, defined as the proportion of patients with no histological evidence of residual invasive tumor cells in the breast (ypT0/is). Secondary outcomes included total pCR (tpCR) rate, ORR, and the incidence of AEs before surgery. The tpCR rate was defined as the proportion of patients with no residual invasive tumor cells in breast and axillary lymph nodes (ypT0/is ypN0). ORR was defined as the proportion of patients with best response of complete or partial response before surgery as per RECIST 1.1. The AEs were graded according to the National Cancer Institute Common Terminology Criteria for Adverse Events version 5.0.

### Statistical Analysis

Statistical analysis was performed using SPSS 15.0 (SPSS Inc., Chicago, IL, USA). Continuous variables were expressed as median (range), and categorical variables were expressed as frequency (percentage). The 95% CIs of bpCR and tpCR were calculated using the Clopper-Pearson method. The bpCR and tpCR rates were also described in subgroups by hormone receptor (HR) status and different neoadjuvant regimens. HR-positive was defined as positive estrogen receptor (ER) and/or progesterone receptor (PR), and HR-negative was defined as negative ER and PR. Comparisons of bpCR and tpCR rates between subgroups by ER, PR, or HR status were performed using chi-square test or Fisher exact test, where appropriate. *P*<0.05 was considered statistically significant.

## Results

### Patient Characteristics

**Figure 1:**
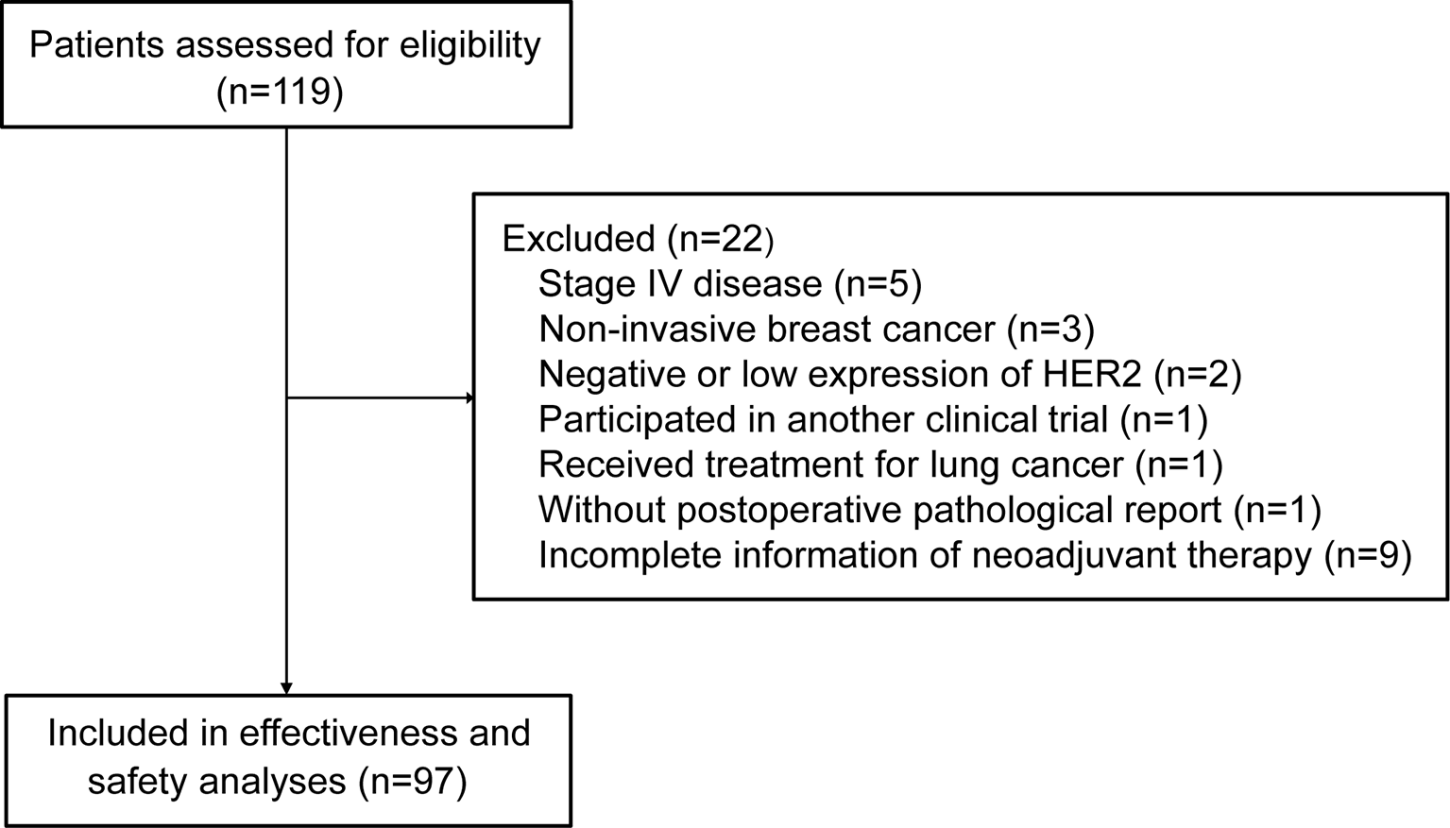
Patient flowchart.

A total of 119 patients were assessed for eligibility. Twenty-two patients did not meet inclusion criteria or met the exclusion criteria, leaving 97 patients included in the analysis (**Figure 1**). The median age was 51 years (range, 24-68). More than half of patients had T2 (59.8%) and N1 (54.6%) disease. Fourteen (14.4%) of 97 patients switched from other anti-HER2 neoadjuvant regimen to pyrotinib-containing regimen (**Table 1**).

**Table 1:** Baseline characteristics.

Characteristics	Patients (N = 97)
Median age (range), years^a^	51 (24-68)
T stage, n (%)	
T1	8 (8.2)
T2	58 (59.8)
T3	20 (20.6)
T4	10 (10.3)
Tx	1 (1.0)
N stage, n (%)	
N0	14 (14.4)
N1	53 (54.6)
N2	13 (13.4)
N3	17 (17.5)
Clinical stage, n (%)	
II	52 (53.6)
III	45 (46.4)
ECOG performance status, n (%)	
0	82 (84.5)
1	15 (15.5)
Hormone receptor status, n (%)	
ER and/or PR positive	45 (46.4)
ER and PR negative	52 (53.6)
Ki-67 level, n (%)	
<30%	19 (19.6)
≥30%	78 (80.4)
Menstrual status, n (%)	
Premenopausal	51 (52.6)
Menopausal	39 (40.2)
Unknown	7 (7.2)
Pathological grading, n (%)	
I	2 (2.1)
II-III	54 (55.7)
Unknown	41 (42.3)
Switching from other anti-HER2 neoadjuvant regimens, n (%)	14 (14.4)
Due to intolerable toxicity	3 (3.1)
Due to poor response	11 (11.3)

ECOG, Eastern Cooperative Oncology Group; ER, estrogen receptor; PR, progesterone receptor; HER2, human epidermal growth factor receptor 2.

a Three patients had missing age.

### Treatment Exposure

Among the 97 patients, 30 (30.9%) received taxane/carboplatin/trastuzumab plus pyrotinib (TCbH+Py), 29 (29.9%) received anthracycline/cyclophosphamide followed by taxane/trastuzumab plus pyrotinib (AC-TH+Py) or taxane followed by anthracycline/cyclophosphamide/trastuzumab plus pyrotinib (T-ACH+Py), 23 (23.7%) received taxane/trastuzumab plus pyrotinib (TH+Py), and 15 (15.5%) received other neoadjuvant regimens (**Table 2**). Twenty-one (70.0%) of 30 patients with TCbH+Py received standard 6-cycle treatment. Twenty-seven (97.2%) of 29 patients with AC-TH+Py or T-ACH+Py received standard 8-cycle treatment. Of 23 patients with TH+Py, 4 (17.4%) received standard 4-cycle treatment, and 17 (73.9%) received treatment for more than 4 cycles (one for 5 cycles and 16 for 6 cycles). Eighty-one (83.5%) of 97 patients had available data of initial dose for pyrotinib. Among them, 66 (81.5%) patients received pyrotinib at an initial dose of 400 mg, and 15 (18.5%) at 320 mg. Fifty-seven (70.4%) patients received 400 mg pyrotinib without dose reductions throughout the neoadjuvant therapy period.

**Table 2:** Neoadjuvant therapy regimens (N = 97).

Regimens	Number of patients (%)	Median treatment cycles (range)
Dual-target		
TCbH+Py	30 (30.9)	6 (1-6)
AC-TH+Py or T-ACH+Py	29 (29.9)	8 (6-8)
TH+Py	23 (23.7)	6 (1-6)
ACH+Py	2 (2.1)	4 (4-4)
AI+H+Py	1 (1.0)	6
ATH+Py	1 (1.0)	1
N+Pb+H+Py	1 (1.0)	2
OFS+AI+H+Py	1 (1.0)	2
TCH+Py	1 (1.0)	4
AC+Py, then switched to TCbHP^a^	1 (1.0)	8
TCbH+Py, then switched to THP^b^	1 (1.0)	5
Single-target or triple-target		
OFS+EC+Py-T+Py	1 (1.0)	8
Py+X	1 (1.0)	6
T+Py+X	1 (1.0)	6
TC+Py	1 (1.0)	6
TCb+Py	1 (1.0)	6
TCbHP+Py	1 (1.0)	4

T, taxane (docetaxel, albumin-bound paclitaxel, or paclitaxel); Cb, carboplatin; H, trastuzumab; Py, pyrotinib; A, anthracycline (epirubicin, pirarubicin, or doxorubicin); C, cyclophosphamide; AI, aromatase inhibitor (anastrozle or exemestane); N, vinorelbine; Pb, cisplatin; OFS, ovarian function suppression (goserelin); X, capecitabine; P, pertuzumab.

a This patient received 2 cycles of AC+Py regimen, then switched to 6 cycles of TCbHP regimen for unknown reason.

b This patient received 2 cycles of TCbH+Py regimen, then switched to 3 cycles of THP regimen because of intolerance to Py.

### Pyrotinib-Containing Neoadjuvant Therapy Outcomes

After pyrotinib-containing neoadjuvant therapy, 22 (22.7%) of 97 patients had a complete response, and 63 (64.9%) had a partial response, with an ORR of 87.6% according to RECIST 1.1. Five patients had stable disease, and seven patients were not evaluable due to no imaging assessment after baseline.

**Figure 2:**
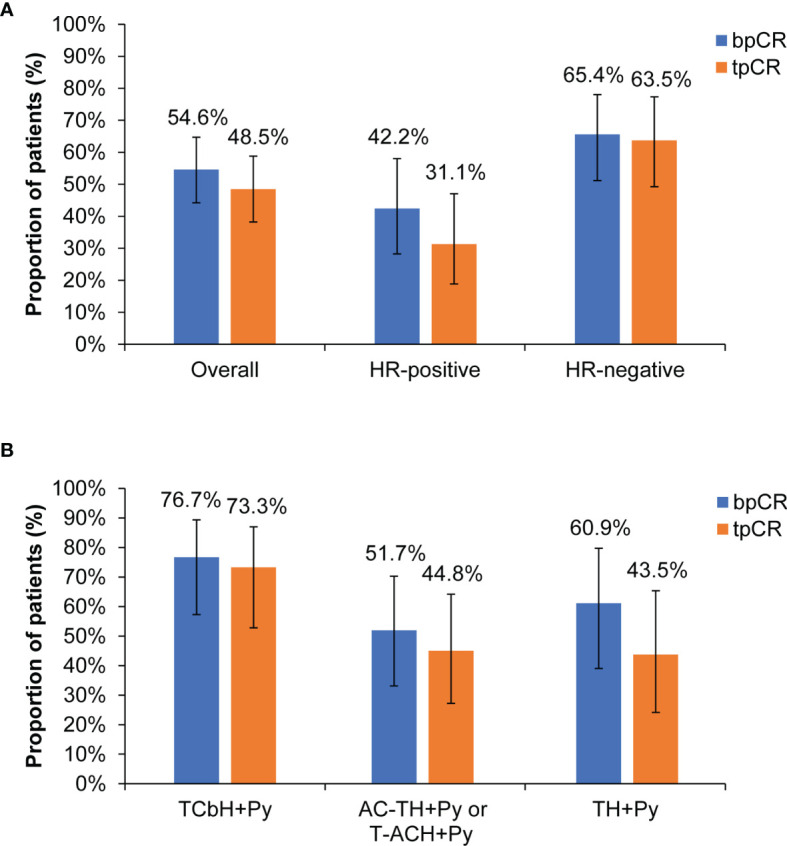
Pathological response. **(A)** Overall population and subgroups by HR status. **(B)** Subgroups by different neoadjuvant regimens. bpCR, breast pathological complete response; tpCR, total pathological complete response; HR, hormone receptor; T, taxane (docetaxel, nanoparticle albumin-bound paclitaxel, or paclitaxel); Cb, carboplatin; H, trastuzumab; Py, pyrotinib; A, anthracycline (epirubicin, pirarubicin, or doxorubicin); C, cyclophosphamide.

After pyrotinib-containing neoadjuvant therapy and surgery, 53 (54.6% [95% CI, 44.2%-64.7%]) patients had pCR in breast, and 47 (48.5% [95% CI, 38.2%-58.8%]) had pCR in both breast and lymph nodes (**Figure 2A**). Among 45 patients with HR-positive disease, the bpCR and tpCR rates were 42.2% (95% CI, 28.0%-57.8%) and 31.1% (95% CI, 18.6%-46.8%), respectively. The bpCR and tpCR rates were 65.4% (95% CI, 50.8%-77.7%) and 63.5% [95% CI, 48.9%-76.0%] in 52 patients with HR-negative disease, respectively (**Figure 2A**). ER, PR, or HR status correlated with tpCR (*P*<0.05), HR status correlated with bpCR (*P*=0.022; **Table 3**). Of 13 patients with ER-positive/PR-negative disease, 61.5% (8/13) had bpCR and 53.8% (7/13) had tpCR. For four patients with ER-negative/PR-positive disease, three (75.0%) had bpCR and tpCR.

**Table 3:** Comparisons of bpCR and tpCR rates between subgroups by ER, PR, or HR status.

Characteristics	bpCR, n (%)	Non-bpCR, n (%)	*P*	tpCR, n (%)	Non-tpCR, n (%)	*P*
ER status			0.069			0.030
Positive (n = 41)	18 (43.9)	23 (56.1)		13 (31.7)	28 (68.3)	
Negative (n = 56)	35 (62.5)	21 (37.5)		34 (60.7)	22 (39.3)	
PR status			0.052			0.005
Positive (n = 32)	13 (40.6)	19 (59.4)		9 (28.1)	23 (71.9)	
Negative (n = 65)	40 (61.5)	25 (38.5)		38 (58.5)	27 (41.5)	
HR status			0.022			0.002
ER and/or PR positive (n = 45)	19 (42.2)	26 (57.8)		14 (31.1)	31 (68.9)	
ER and PR negative (n = 52)	34 (65.4)	18 (34.6)		33 (63.5)	19 (36.5)	

bpCR, breast pathological complete response; tpCR, total pathological complete response; ER, estrogen receptor; PR, progesterone receptor; HR, hormone receptor.

For subgroups by different neoadjuvant regimens, the bpCR rate was 76.7% (95% CI, 57.3%-89.4%) in 30 patients with TCbH+Py, 60.9% (95% CI, 38.8%-79.5%) in 23 patients with TH+Py, and 51.7% (95% CI, 32.9%-70.1%) in 29 patients with AC-TH+Py or T-ACH+Py. The tpCR rate was 73.3% (95% CI, 53.8%-87.0%) in 30 patients with TCbH+Py, 44.8% (95% CI, 27.0%-64.0%) in 29 patients with AC-TH+Py or T-ACH+Py, and 43.5% (95% CI, 23.9%-65.1%) in 23 patients with TH+Py (**Figure 2B**).

### Safety

Treatment-related AEs (TRAEs) before surgery occurred in 92.8% (90/97) of patients. Grade ≥3 TRAEs were observed in 35.1% (34/97) of patients. Serious TRAEs were observed in 5.2% (5/97) of patients. The most common TRAEs were diarrhea (62 [63.9%]), decreased hemoglobin (57 [58.8%]), increased alanine transaminase (36 [37.1%]), nausea (34 [35.1%]), decreased neutrophil count (31 [32.0%]), and decreased white blood cell count (30 [30.9%]). The most frequent grade 3 or 4 TRAEs included diarrhea (15 [15.5%]), decreased hemoglobin (nine [9.3%]), and decreased neutrophil count (eight [8.2%]; **Table 4**). There were eight (8.2%) patients having pyrotinib dose reductions due to TRAEs: the dose was reduced to 320 mg for six patients, 240 mg for one patient, and 160 mg for one patient. No treatment-related deaths occurred.

**Table 4:** Treatment-related adverse events before surgery.

Events, n (%)	Patients (N = 97)		
	Any grade	Grade 3	Grade 4
Diarrhea	62 (63.9)	15 (15.5)	0
Hemoglobin decreased	57 (58.8)	9 (9.3)	0
ALT increased	36 (37.1)	2 (2.1)	0
Nausea	34 (35.1)	4 (4.1)	0
Neutrophil count decreased	31 (32.0)	7 (7.2)	1 (1.0)
White blood cell count decreased	30 (30.9)	4 (4.1)	0
Vomiting	28 (28.9)	4 (4.1)	0
AST increased	28 (28.9)	0	0
Hypokalemia	23 (23.7)	2 (2.1)	2 (2.1)
Fatigue	23 (23.7)	2 (2.1)	0
Platelet count decreased	22 (22.7)	0	2 (2.1)
Creatinine increased	13 (13.4)	0	0
Hand-foot syndrome	7 (7.2)	0	0
Hypomagnesemia	7 (7.2)	0	0
Hyponatremia	4 (4.1)	0	0
Rash	2 (2.1)	0	0
Febrile neutropenia	1 (1.0)	0	1 (1.0)
Upper respiratory infection	1 (1.0)	1 (1.0)	0
Weight loss	1 (1.0)	0	0

ALT, alanine transaminase; AST, aspartate transaminase.

## Discussion

To identify the effectiveness and safety of pyrotinib-containing regimens as neoadjuvant therapy for patients with HER2-positive early or locally advanced breast cancer in the real-world setting, we here made a multicenter retrospective analysis. Lapatinib, neratinib, and pyrotinib are three TKIs investigated in the neoadjuvant setting for patients with HER2-positive breast cancer. Lapatinib is a reversible TKI blocking HER1 and HER2, while pyrotinib is an irreversible pan-ErbB receptor TKI targeting HER2, HER1 and HER4, with similar molecular structure to neratinib (23). In a phase 1 clinical trial, pyrotinib plus capecitabine was well-tolerated and demonstrates promising antitumor activity in patients with HER2-positive metastatic breast cancer (24). Pyrotinib in combination with capecitabine had a significantly higher ORR (78.5% vs. 57.1%, P=0.01) and longer progression-free survival (18.1 months vs. 7.0 months, P<0.001) compared with lapatinib plus capecitabine in a phase 2 study (25). Results from the phase 3 PHOEBE study confirmed that pyrotinib plus capecitabine could lead to progression-free survival benefit compared with lapatinib plus capecitabine (12.5 months [95% CI, 9.7-not reached] vs. 6.8 months [95% CI, 5.4-8.1]; hazard ratio, 0.39 [95% CI, 0.27-0.56]; P<0.0001) in HER2-positive metastatic breast cancer who had previously received anthracycline or taxane chemotherapy (19). Pyrotinib has the advantages of stability, safety, and good tolerance, with encouraging antitumor effects observed in patients with HER2-positive metastatic breast cancer. However, data on its activity in the neoadjuvant setting are still lacking. We supplemented real-world evidence on the basis of previous phase 2 neoadjuvant trials (20–22). The bpCR rate in 97 patients was 54.6% (95% CI, 44.2%-64.7%), and the tpCR rate was 48.5% (95% CI, 38.2%-58.8%). Grade 3 and 4 TRAEs were observed in 35.1% of patients, with the most common events of diarrhea (15.5%), decreased hemoglobin (9.3%), and decreased neutrophil count (8.2%). The safety profile was acceptable.

Taxane/carboplatin/trastuzumab/pertuzumab (TCbHP) and taxane/trastuzumab/pertuzumab (THP) are two class I recommendations in the 2021 version of Chinese Society of Clinical Oncology breast cancer guideline, while other regimens based on anti-HER2 monoclonal antibody and taxane (such as anthracycline/cyclophosphamide followed by taxane/trastuzumab/pertuzumab [AC-THP]) are class II recommendations (3, 4). The National Comprehensive Cancer Network guideline recommended TCbHP as preferred dual anti-HER2 regimens, and recommended the use of AC-THP and taxane followed by anthracycline/cyclophosphamide/trastuzumab/pertuzumab (T-ACHP) in certain circumstances (6). In our study, 84.5% of patients received the common combinations with chemotherapy plus dual HER2-targeted blockade, including 30 patients with TCbH+Py, 29 with AC-TH+Py or T-ACH+Py, and 23 with TH+Py. The selection of neoadjuvant therapy regimens was in accordance with the guidelines (3, 4, 6). Sixty-nine (84.1%) of 82 patients with these common regimens completed the full course of neoadjuvant therapy, including 17 (73.9%) of 23 patients with TH+Py receiving more than standard 4 cycles. The appropriate selection of neoadjuvant therapy regimens and good compliance might contribute to the high pCR rate. This also reflects a standardized clinical practice environment in China.

In the present study, the tpCR rate in patients with TCbH+Py was 73.3%, higher than docetaxel/carboplatin/trastuzumab/pertuzumab (55.7%) in the KRISTINE study (9) and docetaxel/carboplatin/trastuzumab/lapatinib (51.7%) in the TRIO-US B07 study (15). Patients who received TH+Py had a consistent tpCR rate with paclitaxel/trastuzumab/lapatinib in the NeoALTTO study (43.5% vs. 46.8%) (12). The tpCR rate in patients with AC-TH+Py or T-ACH+Py was 44.8%, similar with paclitaxel/trastuzumab/neratinib followed by doxorubicin/cyclophosphamide (50.0%) in the NSABP FB-7 study (16), and paclitaxel followed by fluorouracil/epirubicin/cyclophosphamide/trastuzumab/lapatinib (46.7%) in the CHER-LOB study (26). A previous phase 2 neoadjuvant trial of pyrotinib also showed a high tpCR rate with epirubicin/cyclophosphamide/pyrotinib followed by docetaxel/trastuzumab/pyrotinib (73.7%) (20), but the efficacy might be exaggerated due to the limited sample size (n=19; **Table 5**). Although the cross-study comparisons need to be interpreted in cautions, and the number of patients with different neoadjuvant regimens was small, these results indirectly suggest that trastuzumab plus pyrotinib as dual HER2-targeted blockade is feasible when combined with chemotherapy as neoadjuvant therapy in the real-world setting.

**Table 5:** Pathological response results from current published neoadjuvant trials of tyrosine kinase inhibitor in HER2-positive breast cancer.

Study	Treatment	tpCR rate, n/N (%)			bpCR rate, n/N (%)		
		HR-positive	HR-negative	Total	HR-positive	HR-negative	Total
NSABP FB-7 (16)	TH+Ne-AC	7/23 (30.4)	14/19 (73.7)	21/42 (50.0)	NA	NA	22/42 (52.4)
NeoALTTO (12)	TH+L	NA	NA	68/145 (46.8)	32/77 (41.6)	46/75 (61.3)	78/152 (51.3)
CALGB 40601 (13)	TH+L	28/69 (40.6)	32/47 (68.1)	60/116 (51.7)	28/69 (40.6)	37/47 (78.7)	65/116 (56.0)
NSABP B-41 (14)	AC-TH+L	59/108 (54.6)	44/63 (69.8)	103/171 (60.2)	60/108 (55.6)	46/63 (73.0)	106/171 (62.0)
CHER-LOB (27)	Td-FuECH+L	10/28 (35.7)	10/17 (58.8)	21/45 (46.7)	NA	NA	NA
TRIO-US B07 (15)	TdCbH+L	14/34 (41.2)	16/24 (66.7)	30/58 (51.7)	NA	NA	NA
GBG-70 (28)	H+Af-TH+Af-ECH	20/46 (43.5)	12/19 (63.2)	32/65 (49.2)	NA	NA	36/65 (55.4)
Xuhong et al. (20)	EC+Py-TdH+Py	7/11 (63.6)	7/8 (87.5)	14/19 (73.7)	NA	NA	NA
Zhong et al. (21)	AbTH+Py	3/8 (37.5)	9/13 (69.2)	12/21 (57.1)	NA	NA	NA
Panphila (22)	TdCbH+Py	19/47 (40.4)	19/22 (86.4)	38/69 (55.1)	NA	NA	40/69 (58.0)

HR-positive was defined as positive estrogen receptor and/or progesterone receptor, and HR negative was defined as negative estrogen receptor and progesterone receptor. HER2, human epidermal growth factor receptor 2; T, paclitaxel; H, trastuzumab; Ne, neratinib; A, doxorubicin; C, cyclophosphamide; L, lapatinib; Td, docetaxel; Fu, fluorouracil; E, epirubicin; Cb, carboplatin; Af, afatinib; NA, not available; Py, pyrotinib; AbT, albumin-bound paclitaxel.

Patients with HR-negative disease accounted for 53.6% of the total population. The bpCR and tpCR rates were 65.4% and 63.5%, respectively, numerically higher than those of patients with HR-positive disease (42.2% and 31.1%). This trend was consistent with previous studies (12–16). Actually, we found that ER status just correlated with tPCR, not bPCR. PR status correlated with both bPCR and tPCR. Compared with PR-positive subgroup, PR-negative patients showed higher bpCR (61.5% vs. 40.6%) and tpCR (58.5% vs. 28.1%) rates with pyrotinib-containing neoadjuvant therapy. PR synthesis requires estrogen and ER, and PR expression is upregulated by ER; thus, ER-positive/PR-positive breast cancer is common (29). PR is also a biomarker used routinely at diagnosis to characterize breast cancer. It participates in molecular subtyping and plays a determining factor in treatment decisions. The absence of PR reflects a nonfunctional ER pathway, which is less responsive to selective ER modulators (30). Our findings suggest that both ER and PR status are potential prognostic factors for tpCR in HER2-positive breast cancer with pyrotinib-containing neoadjuvant therapy. Further investigation is needed.

The incidence of TRAEs in our study was 92.8%, and the most common TRAEs were diarrhea (63.9%), decreased hemoglobin (58.8%), increased alanine transaminase (37.1%), nausea (35.1%), decreased neutrophil count (32.0%), and decreased white blood cell count (30.9%). The AE profile was consistent with previous neoadjuvant trials of pyrotinib (20–22) and the phase 3 results of pyrotinib in advanced breast cancer (19, 31). The incidence of hand-foot syndrome was 7.2%, and only grade 1 events occurred. Capecitabine is a partner of pyrotinib when used in advanced breast cancer, and one of its frequently reported AEs is hand-foot syndrome. However, capecitabine is not a common component of standard neoadjuvant regimens, with only two (2.1%) patients receiving capecitabine in our study. This might explain the low incidence and severity of hand-foot syndrome. In addition, only 35.1% of patients had grade ≥3 TRAEs, mainly grade 3 diarrhea (15.5%). The incidence of serious TRAEs was 5.2%. The overall safety of pyrotinib-containing neoadjuvant therapy was manageable, without new safety signals. Of patients with available data of initial dose for pyrotinib, 70.4% received 400 mg pyrotinib without dose reductions throughout the neoadjuvant therapy period, indicating a good tolerability.

There are some limitations in this multicenter real-world study. First, bias is inevitable due to the retrospective nature. Second, the judgement of AEs needs incorporation of medical records, laboratory test reports, and medical advice. There could be recall bias and missing reports in the real-world setting. Thus, the AE data might be underestimated. Third, some patients could not complete the full course of neoadjuvant therapy due to the impact of coronavirus disease 2019 (COVID-19), leading to reduced effectiveness. Fourth, this study only reported pathological response. The long-term outcomes in patients with HER2-positive breast cancer who received pyrotinib-containing neoadjuvant regimens needs further investigation. Finally, although all patients were treated with pyrotinib, the effectiveness and safety might be attributed to other components as many combination regimens were included.

## Conclusions

Small-molecule TKI pyrotinib as a component of neoadjuvant therapy for patients with HER2-positive early or locally advanced breast cancer shows effectivenesswith manageable toxicity in the real-world setting. Trastuzumab plus pyrotinib may be a novel option of dual HER2-targeted blockade when combined withchemotherapy as neoadjuvant therapy. Our study suggests a trend PR-negative was a significant prognostic factor for tPCR or bPCR in HER2+ breast cancer with pyrotinib-containing neoadjuvant therapy.

## Data Availability Statement

The raw data supporting the conclusions of this article will be made available by the authors, without undue reservation.

## Ethics Statement 

The studies involving human participants were reviewed and approved by The ethics committee of the First Hospital of China Medical University (No. [2021]308). The patients/participants provided their written informed consent to participate in this study.

## Author Contributions

FJ and JL contributed to the study conception and design. FJ, JL, XM, PL, YG, XW, PT, SW, DZ, WY, OW, and JZ contributed to the acquisition of data. XM analyzed and interpreted the data. XM drafted the manuscript. FJ and JL contributed to the critical review and revision of the manuscript. All authors contributed to the article and approved the submitted version.

## Funding

This work was supported by the National Natural Science Foundation of China (No. 81972791). The funders had no role in study design, data collection and analysis, decision to publish, or preparation of the manuscript.

## Conflict of Interest

The authors declare that the research was conducted in the absence of any commercial or financial relationships that could be construed as a potential conflict of interest.

## Publisher’s Note

All claims expressed in this article are solely those of the authors and do not necessarily represent those of their affiliated organizations, or those of the publisher, the editors and the reviewers. Any product that may be evaluated in this article, or claim that may be made by its manufacturer, is not guaranteed or endorsed by the publisher.
