# Supplementary material for: Pyrotinib-Containing Neoadjuvant Therapy in Patients With HER2-Positive Breast Cancer: A Multicenter Retrospective Analysis
Source: Front Oncol. 2022 Apr 7;12:855512. doi: 10.3389/fonc.2022.855512 (PMC9021502; doi:10.3389/fonc.2022.855512)
Supplement: Supplementary file 1 [file Table_1.docx]

**Supplementary Table S1.** Number of patients included in the analysis at each center

| **Center** | **Number of patients** |
| --- | --- |
| The First Affiliated Hospital of Zhengzhou University | 31 |
| The Southwest Hospital of Army Medical University | 19 |
| The First Affiliated Hospital of China Medical University | 9 |
| Affiliated Zhongshan Hospital of Dalian University | 5 |
| Fujian Cancer Hospital | 5 |
| The Affiliated Cancer Hospital of Zhengzhou University & Henan Cancer Hospital | 4 |
| Affiliated Hospital of Hubei University of Medicine & Xiangyang No.1 People’s Hospital | 3 |
| Zhejiang Provincial Hospital of Chinese Medicine | 3 |
| Hubei Cancer Hospital, Tongji Medical College, Huazhong University of Science and Technology | 2 |
| Jiangsu Cancer Hospital & Jiangsu Institute of Cancer Research & The Affiliated Cancer Hospital of Nanjing Medical University | 2 |
| The Second Affiliated Hospital of Wenzhou Medical University | 2 |
| Dalian Municipal Central Hospital | 1 |
| Hanchuan People’s Hospital | 1 |
| Henan Provincial People’s Hospital | 1 |
| Renmin Hospital of Wuhan University, Hubei General Hospital | 1 |
| The Central Hospital of Wuhan, Tongji Medical College, Huazhong University of Science and Technology | 1 |
| The First Affiliated Hospital of Dalian Medical University | 1 |
| The First Affiliated Hospital of Soochow University | 1 |
| The First People’s Hospital of Lianyungang | 1 |
| Tongji Hospital, Tongji Medical College, Huazhong University of Science and Technology | 1 |
| Xiamen Maternal and Child Health Hospital | 1 |
